# Soil-Applied Imidacloprid Translocates to Ornamental Flowers and Reduces Survival of Adult *Coleomegilla maculata*, *Harmonia axyridis*, and *Hippodamia convergens Lady Beetles, and Larval Danaus plexippus and Vanessa cardui* Butterflies

**DOI:** 10.1371/journal.pone.0119133

**Published:** 2015-03-23

**Authors:** Vera Krischik, Mary Rogers, Garima Gupta, Aruna Varshney

**Affiliations:** 1 Department of Entomology, University of Minnesota, St. Paul, Minnesota, United States of America; 2 Department of Horticultural Science, University of Minnesota, St. Paul, Minnesota, United States of America; 3 Department of Zoology, Panjab University, Chandigarh, India; Federal University of Viçosa, BRAZIL

## Abstract

Integrated Pest Management (IPM) is a decision making process used to manage pests that relies on many tactics, including cultural and biological control, which are practices that conserve beneficial insects and mites, and when needed, the use of conventional insecticides. However, systemic, soil-applied neonicotinoid insecticides are translocated to pollen and nectar of flowers, often for months, and may reduce survival of flower-feeding beneficial insects. Imidacloprid seed-treated crops (0.05 mg AI (active ingredient) /canola seed and 1.2 mg AI/corn seed) translocate less than 10 ppb to pollen and nectar. However, higher rates of soil-applied imidacloprid are used in nurseries and urban landscapes, such as 300 mg AI/10 L (3 gallon) pot and 69 g AI applied to the soil under a 61 (24 in) cm diam. tree. Translocation of imidacloprid from soil (300 mg AI) to flowers of *Asclepias curassavica* resulted in 6,030 ppb in 1X and 10,400 ppb in 2X treatments, which are similar to imidacloprid residues found in another plant species we studied. A second imidacloprid soil application 7 months later resulted in 21,000 ppb in 1X and 45,000 ppb in 2X treatments. Consequently, greenhouse/nursery use of imidacloprid applied to flowering plants can result in 793 to 1,368 times higher concentration compared to an imidacloprid seed treatment (7.6 ppb pollen in seed- treated canola), where most research has focused. These higher imidacloprid levels caused significant mortality in both 1X and 2X treatments in 3 lady beetle species, *Coleomegilla maculata*, *Harmonia axyridis*, and *Hippodamia convergens*, but not a fourth species, *Coccinella septempunctata*. Adult survival were not reduced for monarch, *Danaus plexippus* and painted lady, *Vanessa cardui*, butterflies, but larval survival was significantly reduced. The use of the neonicotinoid imidacloprid at greenhouse/nursery rates reduced survival of beneficial insects feeding on pollen and nectar and is incompatible with the principles of IPM.

## Introduction

Integrated Pest Management (IPM) is a decision making process used to manage pests that relies on many tactics, including cultural control and biological control, which are practices that conserve beneficial insects and mites, and when needed, the use of conventional insecticides [1,2]. When systemic neonicotinoid insecticides were first registered their use was embraced in IPM due to their low mammalian toxicity [3,4] and the use of soil applications, thereby reducing the nontarget effects on beneficial insects from foliar spraying. However, after spraying a contact, foliar insecticide, new flowers that open will not contain residues. In contrast, systemic insecticides applied to the soil remain in the plant and are expressed in pollen and nectar for a much longer duration [4,5–13].

The neonicotinoid insecticide imidacloprid was registered in 1994 and presently is the second most widely used agrochemical in the world [14]. Systemic, neonicotinoid insecticides have continued to be registered, with active ingredients thiamethoaxam, clothianidin, dinotefuran, and sulfoxaflor [15], all of which are translocated to pollen and nectar and are highly toxic to bees [16]. In the U.S., over 907,185 kg of imidacloprid, clothianidin, and thiamethoxam are used on 58 million ha of 178 million ha of cropland [17].

Recently, the translocation of systemic neonicotinoid insecticides into pollen and nectar has been suggested as one of the many factors contributing to the decline in honey bees and bumble bees [18,12,13]. In 2013 the European Union’s (EU) European Food Safety Authority (EFSA) restricted the use of neonicotinoid insecticides on flowering plants for two years to identify the risk that systemic neonicotinoid insecticides caused to bees. The EFSA review paper on the risk of neonicotinoid insecticides identified a deficiency of studies on residue in crops and landscape plants [16].

Neonicotinoid insecticide residues in pollen and nectar differ widely depending on the amount that is applied to crops and landscapes. Seed treatments result in relatively low levels, less than 10 ppb in pollen and nectar from an application of 1.2 mg AI imidacloprid on corn seed or 0.05 mg AI on canola seed (Gaucho, BayerCropScience, Research Triangle Park, NC) [13,16,19–21]. Imidacloprid residue in pollen from seed-treatments was 4.4 to 7.6 ppb in canola, 3 ppb in sunflower and 3.3 ppb in maize [16,19,22,23]. Imidacloprid residue in nectar from seed-treatments was 0.8 ppb in canola and 1.9 ppb in sunflower [22,23]. Thiamethoxam and clothianidin seed treatments also resulted in low levels in pollen and nectar. Thiamethoxam residue in corn pollen was 1 to 7 ppb, in canola pollen was 1 to 3.5 ppb, and in canola nectar was 0.6 to 2.4 ppb [24]. In numerous studies, seed treatments of imidacloprid and clothianidin did not result in residues in pollen or nectar at sufficient levels to reduce honey bee colony health [13,16,24–27]. A four year study on thiamethoxam seed-treatments on corn and canola reported no adverse effects on honey bee colony health (mortality, foraging behavior, colony strength, colony weight, brood development, and food storage levels) and overwintering [24].

The imidacloprid field crop rate is 4 mg/sq ft (4 mg/939 sq cm) (AdmirePro, Gaucho, BayerCropScience, Research Triangle Park, NC) which is a higher rate than what is applied to seed treatments and should result in higher residue in flowering crops. In squash, residues in pollen were 14.7 ppb for imidacloprid and 12.9 ppb for thiamethoxam and in nectar were 10.3 ppb for imidacloprid and 11.6 ppb for thiamethoxam [28]. In pumpkin, residues in pollen were 31.8 ppb for imidacloprid, 34.7 ppb for dinotefuran, and 25.2 ppb for thiamthoxam and in nectar were 9.1 ppb for imidacloprid, 7.0 ppb for dinotefuran, and 4.3 ppb for thiamethoxam, with maximums of 122 ppb in pollen and 18 ppb in nectar [29]. There are currently no field studies that investigate these higher levels of neonicotinoid insecticides on bee foraging and survival.

In ornamental flowering plants grown in greenhouses, a soil application of imidacloprid is 300 mg AI for a 3 gallon (10 L) pot (Marathon 1%G, Olympic Horticultural Products, Mainland, PA; or Bayer Advanced Tree and Shrub, Bayer CropScience, Research Triangle Park, NC). This is a 250 times higher rate on pots when compared to a seed treatment rate on corn (1.2 mg AI /seed), and a 75 times higher rate when compared to a field crop rate (4 mg AI/sq ft) (4 mg/939 sq cm). In trees, a soil surface drench under the canopy permits 67 g AI imidacloprid for a 61cm (24 in) diameter at breast height (DBH) tree, and 45 g AI imidacloprid for a 41 cm (16 in) DBH tree. If we calculate the area under a 61cm tree to be 10 sq ft, then the amount of imidacloprid applied is 67 g/10 sq ft or 6,700 mg/sq ft (6,700 mg/939 sq cm) compared to 4 mg/sq ft (4 mg/939 sq cm) in agriculture, a 1,675 times greater amount. If these higher amounts of insecticide used on trees in landscapes are translocated to pollen and nectar of the tree, as well as through the soil to nearby flowers, then bees and other beneficial insects feeding on the flowers may be negatively impacted.

Indeed, Bayer’s research on imidacloprid translocation from soil to flowers of landscape plants found very high residues. Dogwood, *Cornus mas*, flowers at 17 months after application contained 1,038–2,816 ppb imidacloprid[10]. Other studies by Bayer found residues of 27–850 ppb in rhododendron flowers at 6 months after application and residues of 19 ppb at 3 to 6 years after application [6,7]; residues of 66–4,560 ppb in serviceberry, *Amelanchier* spp., flowers at 18 months after application; residues of 1,038–2,816 ppb in dogwood, *Cornus mas*, flowers at 17 months after application; and residues of 5 ppb in horse chestnut, *Aesculus hippocastanum*, flowers at 18 months after application [5,8–10]. The California Department of Pesticide Regulation reported dead bumble bees containing 146 ppb imidacloprid residue when little leaf linden, *Tilia cordata*, were treated with a soil drench of imidacloprid at a golf course for two consecutive years [30].

There are currently very few published studies investigating imidacloprid residue in flowers from soil-applied greenhouse/nursery rates and imidacloprid’s impact on survival of pollen and nectar feeding beneficial insects, such as lady beetles and butterflies. Our objectives were: (1) determine the imidacloprid residue in flowers of *Asclepias curassavica* treated with1X and 2X label rates of soil-applied imidacloprid; (2) determine the effects of residue in flowers on survival of four species of adult lady beetles, *Coleomegilla maculata*, *Coccinella septempunctata*, *Harmonia axyridis*, and *Hippodamia convergens*; (3) determine the effects of residue in flowers on adult survival, fecundity, and egg hatch of free-ranging adult monarch, *Danaus plexippus*, and painted lady, *Vanessa cardui*, butterflies; (4) determine the effects of force-fed imidacloprid (0, 15 and 30 ppb) in 30% sugar syrup on adult survival, fecundity, and egg hatch of two species of butterflies; and (5) determine the effects of soil-applied imidacloprid on larval survival of two species of butterflies.

## Methods

### Plants Used for Residue Analysis

Mexican milkweed, *Asclepias curassavica*, has small, open flowers that produce large amounts of nectar that attract beneficial insects [31]. Plugs of Mexican milkweed were purchased from North Creek Nurseries (Landenburg, PA), Rush Creek Growers (Spring Valley, WI), and Michell’s (King of Prussia, PA). Two plugs were planted in 25-cm-diameter black plastic pots (Belden Plastics, Roseville, MN) containing Sunshine SB 500 Universal growing media (Sun Gro Horticulture, Bellevue, WA). Plants were watered daily, and fertilized weekly with a dilute concentration (1g/100ml) of Peter’s general purpose water soluble fertilizer (20%N- 8%P_2_O_5_–16%-K_2_O) (Allentown, PA). Imidacloprid was applied to the soil approximately 3 weeks prior to the start of the experiment to allow time for translocation (1X treatment, 300 AI mg/pot, and 2X treatment, 600 AI mg/pot, Marathon 1%G, Olympic Horticultural Products, Mainland, PA). A second imidacloprid soil application was made 7 months after the first application.

### Residue Analysis

In October of 2007, Enviro-Test Laboratory, ALS Laboratory Group, Edmonton, Canada analyzed Mexican milkweed flowers for determination of the amount of imidacloprid and its hydroxy and olefin metabolites. For residue analysis, each sample was added to 1 ml of water in a 50 ml culture tube, placed in an ultrasonic bath for 2 min, then placed on a wrist shaker for 2 hrs, filtered, partitioned with dichloromethane, filtered, and evaporate to dryness. The residue was dissolved in 20% acetonitrile/0.1% acetic acid and brought to 1 ml, frozen, and then extracted with acetonitrile and concentrated with a rotovaporator. The samples were then analyzed by Liquid Chromatography-Mass Spectrometry LC/MS (PE Sciex API 3200 or 4000 Q-trap system) with variant solvent delivery system and Agilent Automatic Sample Injector. The operating conditions were a YMC-ODS-AM column, 5 μm particle size, 40°C, mobile phase A 0.1% acetic acid in water and mobile phase B 0.1% acetic acid in acetonitrile, flow rate 0.5 ml/min, and injection volume 15 μl. Gradient was 0 min 90% A, 10% B; 6.5 min 30% A, 70% B; 8.0 min 50% A, 50% B; 13 min 90% A, 10% B. The standards were received from Bayer CropSciences (Research Triangle Park, NC) (imidacloprid lot no. 0625200305, purity 99.2%; hydroxy lot no. 072620061, purity 96.8%; olefin lot no. 12192000301, purity 79.8%). The spiking standards were prepared in 20% acetonitrile/0.1% acetic acid. Samples were fortified with imidacloprid, hydroxy, and olefin at 0.05 and 0.10 ppm. Retention time was 7.75 min for imidacloprid (mass transition 256 to 209), 7.36 min for hydroxy (mass transition 272 to 225), and 7.24 min for olefin (mass transition 254 to 207). The limit of quantification for imidacloprid, hydroxy, and olefin was 0.005 ppm based on a 1 g sample and final volume of 1 ml. The average recovery of imidacloprid, hydroxy, and olefin was 95%, 74%, and 96% respectively at 0.05, 0.10, and 15 ppm.

After the residue analysis was done in 2007 at the Canadian lab, the samples were shipped in October 2011 and analyzed by the USDA AMS Gastonia, NC lab using the Canadian method and the USDA standard method. The Quenchers-NSL (National Standard Lab) extraction method was used and the sample was then analyzed by liquid chromatography coupled with tandem mass spectrometry detection (LC/MS/MS) [32–34]. The LC system that was used was an Agilent 1200 Rapid Resolution system with a Zorbax SB-C18 Rapid Resolution HT 2.1650 mm and a1.8 micron column using a 3 ul injection with the gradient going from 5% methanol in water to 100% methanol at 0.45 mL/min. The LC was coupled to a Thermo-LTQ, a linear ion trap mass spectrometer. The system was operated in the positive ion electrospray mode, with a unique scan function for each compound allowing for MS/MS monitoring.

Using these extraction and analysis conditions, spiked control samples containing the compounds averaged 95% recovery with detection limits ranging from 1 ppb to 50 ppb depending on compound, matrix and the amount of sample available for testing. A process control was added to each sample to monitor the effectiveness of the extraction procedure. The average recovery for this compound was 71%. Quantification was performed using external calibration standards prepared from certified standard reference material.

### Lady Beetles: Adult Survival

Both seven-spotted lady beetle (*Coccinella septempunctata*, Crookston, MN, 47°47′59.91″N 96°36′28.79″W, July 2007) and pink lady beetle (*Coleomegilla maculata*, British Columbia, CA, 49°15′12″N 123°15′00″W, June 2007) were collected by colleagues from soybeans that were not treated with insecticides. Multicolored Asian lady beetle was collected from an overwintering site in a barn (*Harmonia axyridis*, Rosemount, MN, 44°44′22″N 93°07′33″W, Nov 2007). No specific permissions were required for collecting lady beetles and these lady beetle species are not endangered or protected. In May 2007, convergent lady beetle (*Hippodamia convergens*) was ordered from Rincon-Vitova Insectaries (Ventura, CA).

Beetles were reared in mesh cages (30 cm x 30 cm x 30 cm, BioQuip, Rancho Dominquez, CA) and for protein were fed organic cat food (Wellness canned brand, beef and chicken), apple slices, and artificial bee pollen (Mann Lake, Hakensack, MN) (3–35mm petri dishes/cage). In addition, 4 tubes with water (Aquatube, Syndicate Sales, Kokomo, IN) and 4 tubes with 50% honey-water were provided. Beetles fed freely for at least 3 weeks prior to the bioassays.

Bioassay containers were 10 cm x 2 cm (diameter) Aquapic (Syndicate Sales, Inc., Kokomo, Indiana) water tubes. In the plastic cap of each tube a 0.5 ml centrifuge tube with a hinged plastic cap and pointed end was inserted. A hole was made in the pointed end to accommodate a flower stalk. Flowers were placed in opposite ends of the tube. Water was placed inside the centrifuge tube to keep the flower hydrated. Flowers were changed daily to insure food availability. All bioassay chambers were kept in laboratory incubators and maintained with a 12:12 (L:D) photoperiod at 25°C and 70 to 75% RH.

Three replicate experiments were performed simultaneously for 6 treatments: control flowers-control flowers (C-C), control flowers-1X treated flowers (C-1X), 1X treated flowers-1X treated flowers (1X-1X), control flowers-2X treated flowers (C-2X), and 2X treated flowers-2X treated flowers (2X-2X). Each replicate had 7–10 bioassay tubes/ treatment and each tube contained 8–10 lady beetles. Bioassays were conducted on convergent lady beetle (15 June 2007), seven-spotted lady beetle (24 August 2007), pink lady beetle (1 August 2007), and multicolored Asian lady beetle (1 June 2008).

### Butterfly Colonies

All butterfly studies were performed in the greenhouse with 16:8 (L:D) photoperiod at 20°/16°C. Monarch butterfly (*Danaus plexippus*) pupae were purchased from the Monarchs in the Classroom Laboratory (University of Minnesota, St. Paul, MN) and Gulf Coast Butterfly Co. (Naples, FL). Prior to the start of the experiment, newly emerged adult monarchs were checked for protozoan (*Ophryocystis elektroscirrha*) spores as infection can reduce lifespan [35] and infection is common in laboratory-reared butterflies [36]. Healthy, non-infected monarch butterflies were fed 30% syrup and placed in individual glassine envelopes in an incubator at 11° C and 60–70% RH for a maximum of 1 week, until released in greenhouse cages. Painted lady butterfly (*Vanessa cardui*) pupae were purchased from Clearwater Butterfly Co. (Chuluota, FL). For eclosion, pupae were attached with dress pins thru the terminal attachment fibers to coffee filters that were hung on the sides of mesh cages.

### Butterflies Free-Ranging: Adult Survival, Fecundity, and Egg Hatch

In the greenhouse, monarch and painted lady butterflies were housed in mesh cages (60 x 60 x 120 cm, BioQuip, Rancho Dominguez, CA) that contained 6 to 8t pots of flowering Mexican milkweed that were changed twice a week. Sponges with 30% honey water were attached to cage frames for butterfly feeding to prevent dehydration incase nectar was limited during the heat of the day.

All experiments ran until ≤10% of the initial population remained. Dead adults were frozen and later sex was determined. In monarchs, sex was determined by the presence of scent glands on the hind wings of the males [37] and by identifying male genitalia. In painted ladies, sex was determined by inspection of the forelegs; as female forelegs have spines that are used in drumming during oviposition, while male forelegs are brush-like [38]. Data on fecundity and egg hatch was determined by removing foliage with eggs and placing the eggs on control leaves in 150 mm petri dishes. The number of eggs counted on the host plants was divided by the number of females to determine fecundity and the number of eggs hatched was recorded. Data on fecundity and egg hatch of free-ranging adult monarch butterflies is lacking.

For monarch butterflies, 4 replicate experiments were performed, each consisting of 3 treatments (C, 1X and 2X). Dates of replicate experiments were: Replicate 1 (5 July 2005, soil was treated on 15 June 2005); Replicate 2 (19 August 2005, soil was treated on 29 July 2005); Replicate 3 (12 September 2005, soil was treated on 22 August 2005); and Replicate 4 (17 October 2005, soil was treated on 26 September 2005). For painted lady butterflies, 3 replicate experiments were performed, each consisting of 3 treatments (C, 1X and 2X). Dates of replicate experiments were: Replicate 1 (16 May 2007, soil was treated on 25 April 2007); Replicate 2 and 3 (26 June 2007, soil was treated on 4 June 2007).

### Butterflies Force-Fed Treated Syrup: Adult Survival, Fecundity, and Egg Hatch

The treatments were made by adding 0.15 g (1X, 15 ppb) or 0.30 g (2X, 30 ppb) of analytical grade imidacloprid (99% purity, ChemServices, West Chester, PA) to 30% sugar syrup. Butterflies were force-fed every 2 days by weighing them down using a 1 cm hex nut on a 96-well tissue culture plate (Linbro/Titertek, Flow Laboratories, Mclean, VA) that held approximately 0.25 ml of syrup. The butterfly proboscis was extended with a pin, and butterflies were allowed to drink from the syrup until they withdrew 3 times. After feeding, butterflies were placed in cages in the greenhouse on host plants with the flowers removed to prevent supplemental feeding. Experiments ran until ≤10% of the initial population was remaining. Dead adults were frozen and later sex was determined as described above. Fecundity and egg hatch were determined as described above.

For monarch butterflies, 4 replicate experiments were performed, each consisting of 3 treatments (C, 1X and 2X). Dates of replicate experiments were: Replicate 1 (19 July 2006); Replicate 2 (28 August 2006); Replicate 3 (22 October 2006); and Replicate 4 (2 March 2007). For painted lady butterflies, 3 replicate experiments were performed, each consisting of 3 treatments (C, 1X and 2X). Dates of replicate experiments were: Replicate 1 (2 March 2007); Replicate 2 (31 March 2007); and Replicate 3 (12 May 2007).

### Plants Used for Larval Feeding

Mexican milkweed was the larval host plant for monarch butterflies. For painted lady butterflies a suitable larval host plant needed to be determined. Painted lady butterflies are polyphagus and larval host plants include thistles (Asteraceae), nettles (Urticaceae), soybean and lupine (Fabaceae), and hollyhock (Malvaceae) [39–41]. The 24 hr host plant preference of first-instar painted lady larvae was studied by cutting 3 discs from 3 leaves and placing the discs in 150 mm petri dishes with 6 larvae (n = 10 discs/host species). We tested larval preference on 6 plants: globe thistle, *Echinops ritro*,; stinging nettle, *Urtica dioica*; soybean, *Glycine max*; lupine, *Lupinus* spp.; hollyhock, *Alcea rosea*; and dandelion, *Taraxacum officinale* (Leitner’s Garden Center, St. Paul, MN; Linder’s, Falcon Heights, MN; Gertens, Inver Grove Heights, MN). Globe thistle, *E*. *ritro*, had the highest total area eaten so it was chosen as the larval host plant.

Larval host plants were grown in the greenhouse. Three plugs of either Mexican milkweed or globe thistle were planted in 25-cm-diameter black plastic pots containing Sunshine SB 500 Universal growing media. Plants were watered as needed, and fertilized weekly with a dilute (1g/100ml) concentration of Peter’s general purpose water soluble fertilizer (20%N- 8%P_2_O_5_–16%K_2_O). Imidacloprid was applied to the soil approximately 3 weeks prior to the start of the experiment to allow time for translocation (1X treatment, 300 mg AI/pot and 2X treatment, 600 mgA/pot, Marathon 1%G, Olympic Horticultural Products, Mainland, PA).

### Butterflies: Larval Survival

Early instar larave were placed on whole intact plants that experienced no prior feeding to evaluate larval survival. For monarch butterflies, 4 replicate experiments were performed for 3 treatments (C, 1X and 2X). For painted lady butterflies, 2 replicate experiments were performed for 3 treatments (C, 1X and 2X). Larval survival was recorded every 3 days until 10% of the larvae remained. For monarchs, dates of replicate experiments and soil treatments were: Replicate 1 (11 Aug 2006, soil treated June 2006); Replicate 2 (15 March 2007, soil treated 9 February 2007); Replicate 3 (18 April 2007, soil treated 9 March); and Replicate 4 (4 May 2007, soil treated 20 April 2007). For painted lady butterflies, dates of replicate experiments and soil treatments were: Replicate 1 and 2 (5 July 2007, soil treated 7 May 2007).

### Statistical Analysis

Survival data for lady beetles and butterflies were analyzed by using a Cox proportional hazards model with a random effect for cage, and stratified by replicate. Pairwise comparisons of the hazard ratios between all treatments were then performed, using a stepwise procedure to correct for multiple comparisons. Differences were considered significant at the 0.05 level. Calculations were performed in R 3.1.0 using the coxme and multcomp packages [42–44]. There was no difference in statistical significance between this method and the original ANOVA analysis.

Survival by day and fecundity data were analyzed by ANOVA and Levene's test to determine homogeneity of variance. If variances were unequal, a Welch test was used and means were compared with Tukey-Kramer MRT. Also, data were analyzed with one-way ANOVA for treatment, replicate, and replicate by treatment interactions using PROC GLM. Means were compared with Tukey-Kramer MRT [45,46]. Comparison of the Canadian and USDA methods of residue analysis was performed with a paired t-test and replicates were combined. Residues of imidacloprid and olefin and hydroxyl metabolites were analyzed by one-way ANOVA, Levene’s test, Welch’s test, and means were compared with Tukey-Kramer MRT.

## Results

### Residue Analysis

Residue data for imidacloprid and olefin metabolite are statistically similar from both labs (ALS Laboratory, Canada, and USDA, Gastonia, NC). The hydroxyl metabolite was found at higher levels with the Canadian method. However, the olefin and hydroxyl metabolites were both small, approximately <20% of the imidacloprid residues (Table 1).

**Table 1:** Imidacloprid, hydroxy, and olefin residue (ppm) extracted from Mexican milkweed, *Asclepias curassavica*, flowers (1 g) that were untreated (C), treated with label rate (1X), or twice label rate (2X) of soil-applied imidacloprid (Marathon 1%G).

USDA-AMS-NSL (ppm)				ALS, Canada (ppm)		
	Imid	5	Olefin	Imid	5	Olefin
		Hydroxy			Hydroxy	
**Replicate 1**						
untreated	0.0121	0	0	0	0	0
untreated	0.0135	0	0	0	0	0
untreated	0.0216	0	0	0	0	0
untreated	5.46	0.208	0.453	3.63	0.338	0.186
**Replicate 2**						
untreated	0.0117	0	0	0	0	0
untreated	0.0060	0	0	0	0	0
untreated	0.0091	0	0	0	0	0
untreated	0.0184	0	0	0.04	0	0
**Replicate 1**						
1X	7.57	0.157	0.386	8.20	0.565	0.405
1X	10.40	0.177	0.495	9.40	0.565	0.390
1X	6.43	0.241	0.471	4.80	0.720	0.500
1X	5.44	0.198	0.410	5.10	0.510	0.360
1X	7.57	0.157	0.386	8.20	0.565	0.405
**Replicate 2**						
1X	8.80	0.527	0.946	24.00	3.400	2.900
1X	22.50	1.470	2.540	26.00	3.500	2.700
1X	32.50	1.590	2.340	34.00	4.600	2.700
**Replicate 1**						
2X	14.70	0.393	0.504	16.00	0.710	0.410
2X	15.60	0.436	0.609	18.00	0.940	0.710
2X	8.02	0.256	0.437	6.40	0.300	0.200
2X	6.93	0.192	0.460	4.60	0.250	0.180
2X	43.30	1.230	2.520	68.00	8.200	4.300
**Replicate 2**						
2X	36.10	1.990	2.520	42.00	6.100	2.700
2X	53.70	2.680	3.140	42.00	5.900	3.200
2X	34.00	1.790	2.110	32.00	4.200	1.900
2X	43.30	1.230	2.520	68.00	8.200	4.300
Paired t-test	1.1087	3.2989	1.6533	—	—	—
n	24	24	24	—	—	—
P	0.2790	0.003	0.1119	—	—	—

Residue analysis methods are from ALS Laboratories, Edmonton, Alberta, CA. and USDA AMS Lab, Gastonia, NC, USA.

Residue analysis on a composite sample of Mexican milkweed flowers (1g) showed imidacloprid residues in flowers at 6.03 ppm in 1X and 10.4 ppm in 2X treatments. A second imidacloprid application 7 months after the first resulted in more than double the imidacloprid residue at 21.67 ppm in 1X and 45.89 ppm in 2X treatments (Table 2). Higher imidacloprid residues from a September soil application may be due to slower vegetative growth rates causing more imidacloprid to concentrate in the flowers rather than leaves. Imidacloprid residue in 1X and 2X treatments were similar to our residue data on buckwheat flowers [47] (Table 3). Residues in pollen and nectar may be different than residue in whole flowers and the correlation needs to be scientifically determined.

**Table 2:** Imidacloprid, hydroxy, and olefin residue (ppm) extracted from Mexican milkweed, *Asclepias curassavica*, flowers (1 g) that were untreated (C), treated with label rate (1X), or twice label rate (2X) of soil-applied imidacloprid (Marathon 1%G).

	Rep 1	Rep 2	Rep 3	Rep 1–3	Rep 1–3….2^nd^ trt 7 mos
	21 d	37 d	51 d	mean21–51 d	mean 234 d
**Imidacloprid (ppm mean ± SE)**					
**untreated**	0.0 ± 0.0c	0.00 ± 0.00b	1.20 ± 1.20c	0.40± 0.40c	0.00 ± 0.00c
**1X**	8.00 ± 0.85b	5.33 ± 0.68a	4.87 ± 0.67b	6.03 ± 1.01b	21.67 ± 2.5b
**2X**	17.00 ± 1.41a	7.33 ± 3.30a	9.07 ± 0.38a	10.40 ± 4.61a	45.89 ± 3.7a
ANOVA	259.20(2,4)	11.39(2,6)	22.72(2,6	25.86(2,22)	79.00(2,24)
*P*	0.0001	0.0091	0.0016	0.001	0.0001
Levene's	0.02(2,4)	7.4702(2,6)	3.79(2,6)	5.60(2,22)	11.22(2, 24)
*P*	0.0000	0.0091	0.0864	0.0107	0.042
Welch's	182.6(2,1.33)	79.59(2,3)	24.8 (2,3.44)	37.23(2,12)	103.36(2,10)
*P*	0.0236	0.0042	0.0090	0.001	0.0001
ANOVA trt	NA	NA	NA	102.60(2,4)	82.94(2,4)
*P trt*				0.0001	0.0001
rep	NA	NA	NA	17.03(2,4)	1.52(2, 4)
*P* rep				0.0001	0.2447
Trt x rep	NA	NA	NA	8.46(2,4)	1.04(2,4)
*P* trt x rep				0.0007	0.4153
**Hydroxy metabolite (ppm mean ± SE)**					
**untreated**	0.00 ± 0.0b	0.00± 0.00a	0.11± 0.11a	0.00 ± 0.00b	0.00 ± 0.0c
**1X**	0.56 ± 0.3a	0.55 ± 0.48a	0.64 ±0.23a	0.61 ± 0.08a	3.07 ± 0.35b
**2X**	0.83 ± 0.16a	0.62 ± 0.16a	0.62 ± 0.06a	0.65 ± 0.10a	5.97 ± 0.36a
ANOVA	67.47(2,4)	4.32(2,6)	4.07(2,6)	19.84(2,22)	102.21(2,24)
*P*	0.0008	0.0688	0.0713	0.0001	0.0001
Levene's	0.00(2,4)	11.62(2,6)	4.49(2,6)	2.40(0.22)	1.07(2, 24)
*P*	0.0000	0.0086	0.0642	0.1142	0.3590
Welch's	66.77(2,1.33)	42.95(2,3)	6.93(2,3.44)	28.21(2,12)	90.86(2,14)
*P*	0.0441	0.0552	0.0632	0.0001	0.0001
trt	NA	NA	NA	18.12(2,4)	118.68(2,4)
*P* trt				0.0001	0.0001
rep	NA	NA	NA	0.26(2,4)	1.39(2,4)
*P* rep				0.7744	0.2755
trt x rep	NA	NA	NA	0.44(2,4)	1.77(2,4)
*P* trt x rep				0.7788	0.1781
**Olefin metabolite (ppm mean ± SE)**					
**untreated**	0.0 ± 0.0b	0.08 ± 0.1b	0.06± 0.06b	0.03 ± 0.02b	0.0 ± 0.00c
**1X**	0.39 ± 0.02a	0.33 ± 0.2a	0.31 ± 0.06a	0.37 ± 0.03a	2.37 ± 0.31b
**2X**	0.58 ± 0.23a	0.42±0.08ab	0.48 ± 0.00a	0.45 ± 0.07a	3.48 ± 0.30a
ANOVA	15.85(2,4)	6.96(2,6)	18.84(2,6)	27.37(2,22)	50.42(2,24)
*P*	0.0125	0.0273	0.0026	0.0001	0.0001
Levene's	0.09(2,4)	10.80(2,6)	1.84(2,6)	3.18(0.22)	1.36(2, 24)
*P*	0.0001	0.0103	0.2389	0.0613	0.2763
Welch's	508.99(2,1.33)	44.96(2,3)	15.6(2,3.44)	46.62(2,12)	38.42(2,14)
*P*	0.0120	0.0088	0.0206	0.0001	0.0001
ANOVAtrt	NA	NA	NA	33.07(2,4)	45.07(2, 4)
*P*				0.0001	0.0001
rep	NA	NA	NA	0.68(2,4)	0.35(2,4)
*P*				0.5196	0.7119
trt x rep	NA	NA	NA	1.59(2,4)	0.69(2,4)
*P*				0.2253	0.6082

Residue analysis was performed at ALS Laboratories, Edmonton, Alberta, CA. Means in the same column followed by different letters are significantly different, Tukey-Kramer MRT,α = 0.05.

**Table 3:** Comparison of imidacloprod, hydroxy, and olefin residue (ppm) in Mexican milkweed, *Asclepias curassivica*, and buckwheat, *Fagopyrum esculentum*, flowers (1 g) [47] that were untreated (C), treated with label rate (1X), or twice label rate (2X) of soil-applied imidacloprid (Marathon 1%G).

	untreated	1X	2X
**Imidacloprid ppm (mean ± SE)**			
buckwheat 21 d	0.00 ± 0.00	6.60 ± 1.00	12.30 ± 2.70
milkweed 21 d	0.40 ± 0.40	6.03 ± 1.01	10.40 ± 4.61
2^nd^ trt 7mos	0.00 ± 0.00	21.67±2.45	45.89 ± 3.74
**Hydroxy metabolite ppm (mean ± SE)**			
buckwheat 21 d	0.00 ± 0.00	1.08 ± 0.20	1.94 ± 0.40
milkweed 21 d	0.00 ± 0.00	0.61 ± 0.08	0.65 ± 0.10
2^nd^ trt 7mos	0.00 ± 0.00	3.07 ± 0.35	5.97 ± 0.36
**Olefin metabolite ppm (mean ± SE)**			
buckwheat 21 d	0.00 ± 0.00	0.20 ± 0.10	0.51 ± 0.10
milkweed 21 d	0.03 ± 0.02	0.37 ± 0.03	0.45 ± 0.07
2^nd^ trt at 7mos	0.0 ± 0.00	2.37 ± 0.31	3.48 ± 0.30

### Lady Beetle: Adult Survival

**Fig 1:**
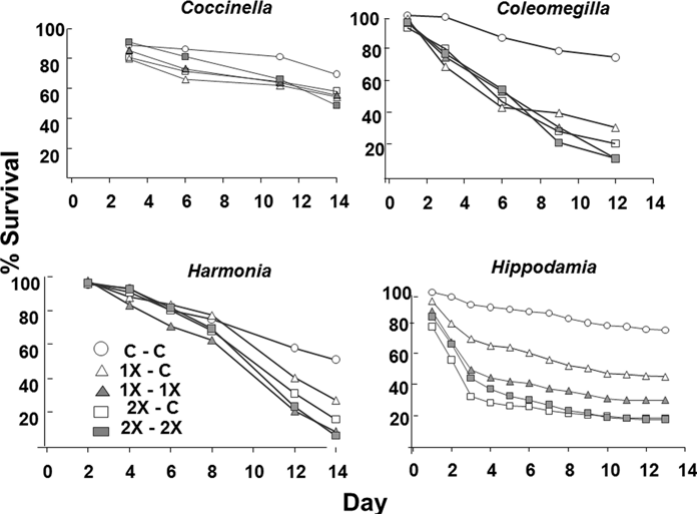
Survival for 14 days of four species of adult lady beetles that were allowed to feed on flowers from Mexican milkweed, *Asclepias curassavica*, plants that were untreated (C), treated with label rate (1X), or twice label rate (2X) rate of soil-applied imidacloprid (Marathon 1%G). Flowers were presented in opposite ends of a tube cage (C-C, 1X-C, 1X-1X, 2X-C, 2X-2X).

**Fig 2:**
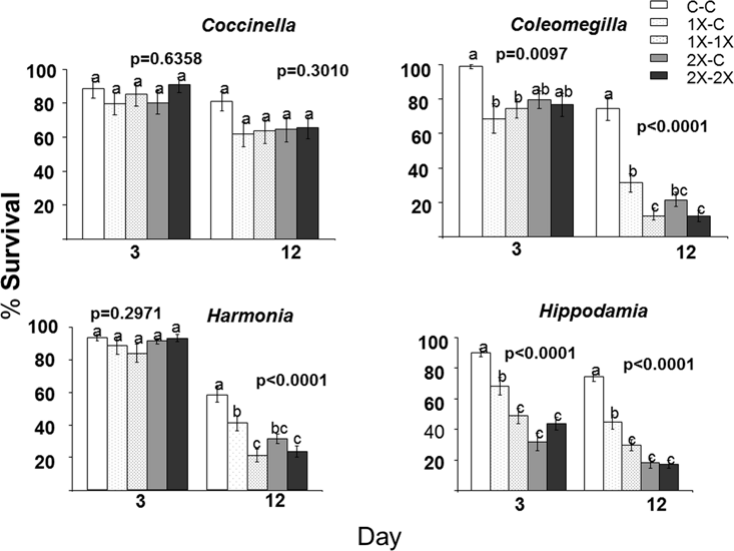
Survival at days 3 and 12 of four species of adult lady beetles that were allowed to feed on flowers from Mexican milkweed, *Asclepias curassavica*, plants that were untreated (C), treated with label rate (1X), or twice label rate (2X) of soil-applied imidacloprid (Marathon 1%G). Flowers were presented in opposite ends of a tube cage (C-C, 1X-C, 1X-1X, 2X-C, 2X-2X).

Imidacloprid significantly reduced survival of 3of 4 species of lady beetles (*Coleomegilla*, *Harmonia*, and *Hippodaemia*) (Fig. 1, Table 4). At day 3, 2 species (*Coleomegilla* and *Hippodaemia*) have significantly lower survival in some treatments compared to controls, while at day 12, 3 species had significantly lower survival in 1X and 2X treatments than controls (Fig. 2, Table 5). It did not appear that beetles avoided feeding on imidacloprid treated flowers, since by day 12 for all 3 beetle species, most 1X-C, 1X-1X, 2X-C, and 2X-2X treatments were not significantly different (Fig. 2, Table 5).

**Table 4:** Survival of four species of adult lady beetles that were fed Mexican milkweed, *Asclepias curassavica*, flowers that were untreated (C), treated with label rate (1X), or twice label rate (2X) of soil-applied imidacloprid (Marathon 1%G).

Treatment	Coef	Exp (coef)	SE (coef)	P value
***Coccinella***				
1X-C / C-C	0.77	2.17	0.43	0.384
1X-1X / C-C	0.63	1.89	0.43	0.5845
2X-C / C-C	0.54	1.72	0.44	0.7298
2X-2X / C-C	1.00	2.73	0.42	0.1211
***Coleomegilla***				
1X-C / C-C	1.89	6.59	0.31	<0.0001
1X-1X / C-C	2.01	7.44	0.30	<0.0001
2X-C / C-C	2.04	7.70	0.31	<0.0001
2X-2X / C-C	2.25	9.48	0.31	<0.0001
***Harmonia***				
1X-C / C-C	0.67	1.95	0.19	0.0036
1X-1X / C-C	1.33	3.80	0.18	<0.0001
2X-C / C-C	0.88	2.40	0.18	<0.0001
2X-2X / C-C	1.12	3.07	0.18	<0.0001
***Hippodamia***				
1X-C / C-C	1.15	3.16	0.21	<0.0001
1X-1X / C-C	1.65	5.19	0.20	<0.0001
2X-C / C-C	2.15	8.55	0.20	<0.0001
2X-2X / C-C	1.91	6.76	0.20	<0.0001

Cox proportional hazards model with a random effect for cage, and stratified by replicate,α = 0.05.

**Table 5:** Survival of four species of adult lady beetles that were fed Mexican milkweed, *Asclepias curassavica*, flowers that that were untreated (C), treated with label rate (1X), or twice label rate (2X) of soil-applied imidacloprid (Marathon 1%G).

	*Coccinella*	*Coleomegilla*	*Harmonia*	*Hippodamia*
**Day 3 (mean ± SE)**				
**C-C**	88.9 ± 5.7a	98.9 ± 1.1a	93.5 ± 1.8a	90.0 ± 2.5a
**1X-C**	79.9 ± 6.7a	68.6 ± 8.5b	88.4 ± 5.0a	68.3 ± 5.6b
**1X-1X**	85.4 ± 6.8a	74.5 ± 5.5b	83.9 ± 5.6a	49.2 ± 4.9c
**2X-C**	80.6 ± 6.8a	79.6 ± 5.1ab	91.3 ± 1.8a	32.1 ± 5.7c
**2X-2X**	90.9 ± 4.5a	76.6 ± 6.6ab	99.3 ± 2.3a	43.8 ± 4.1c
ANOVA	0.6(4,115)	3.5(4,113)	1.2(4,145)	23.2(4,145)
*P*	0.6358	0.0097	0.2971	0.0001
Levene's	0.9(4,115)	9.4(4,113)	6.5(4,145)	8.0(4,145)
*P*	0.4756	0.0001	0.0001	0.0001
Welch's	0.7(4,57)	12.2(4,49)	0.9(4,70)	40.3(4,70)
*P*	0.5856	0.0001	0.4487	0.0001
Model	0.8(14,105)	2.4(14,103)	3.2(14,135)	7.8(14,135)
*P*	0.7156	0.0071	0.0002	<0.0001
Trt	0.6(4,105)	3.7(4,103)	1.4(4,135)	23.9(4,135)
*P*	0.6435	0.0071	0.2516	0.0001
Rep	0.7(2,105)	1.9(2,103)	5.7(2,135)	1.3(2,135)
*P*	0.4847	0.1576	0.0043	0.2849
Trt x Rep	0.8(8,105)	1.8(8,103)	3.6(8,135)	1.5(8,135)
*P*	0.5842	0.0965	0.0009	0.1719
**Day 12 (mean ± SE)**				
**C-C**	81.3 ± 5.7a	74.4 ± 5.8a	58.5 ± 4.6a	74.6 ± 3.1a
**1X-C**	61.8 ± 7.3a	31.4 ± 5.3b	41.1 ± 4.8b	45.0 ± 4.7b
**1X-1X**	63.9 ± 7.5a	12.1 ± 2.6c	21.4 ± 4.1c	30.0 ± 3.6c
**2X-C**	64.6 ± 7.6a	21.5 ± 4.2bc	31.7 ± 3.2bc	18.3 ± 3.5c
**2X-2X**	65.9 ± 7.1a	12.0 ± 3.1c	23.8 ± 3.6c	17.5 ± 2.7c
ANOVA	1.2(4, 115)	33.9(4,113)	13.9(4,145)	44.1(4,145)
*P*	0.0301	0.0001	0.0001	0.0001
Levene's	1.5(4,115)	4.2(4,113)	1.5(4,145)	2.8(4,145)
*P*	0.2032	0.0033	0.1984	0.028
Welch’s	1.6(4,57)	25.7(4,55)	11.8(4,72)	57.5(4,72)
*P*	0.1893	0.0001	0.0001	0.0001
Model	0.65(14,105)	12.1(14,103)	9.5(14,135)	16.6(14,135)
*P*	0.8159	0.0001	0.0001	0.0001
Trt	1.2(4,105)	37.5(4,103)	18.4(4,135)	50.4(4,135)
*P*	0.3271	0.0001	0.0001	0.0001
Rep	0.5(2,105)	1.5(2,103)	22.8(2,135)	2.9(2,135)
*P*	0.6296	0.2345	0.0001	0.0589
Trt x Rep	0.4(8,105)	2.2(8,103)	1.7(8,135)	3.1(8,135)
*P*	0.8963	0.031	0.1166	0.0027

Means in the same column followed by different letters are significantly different, Tukey-Kramer MRT,α = 0.05.

### Butterflies Free-ranging and Force-Fed Treated Syrup: Adult Survival, Fecundity, and Egg Hatch

**Fig 3:**
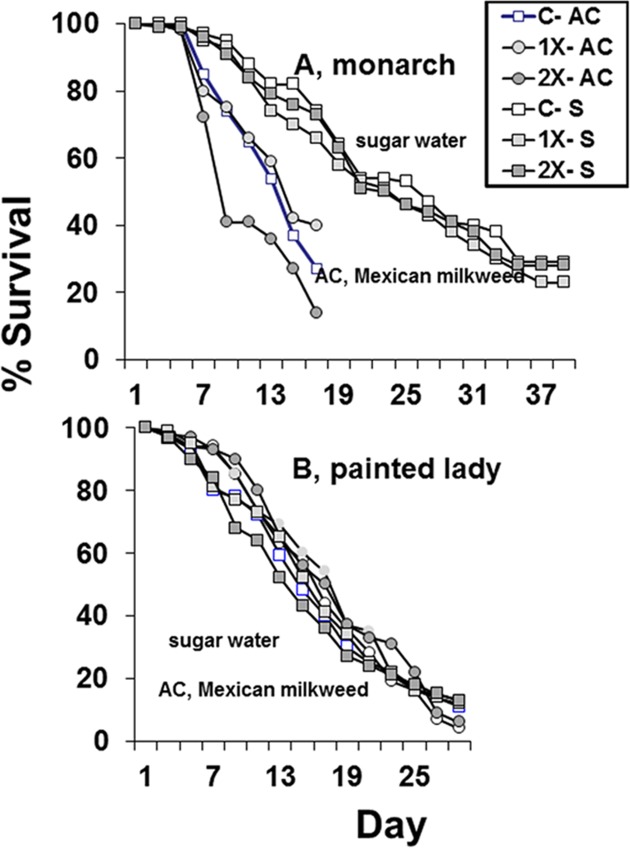
Survival of two species of adult butterflies that were free-ranging and allowed to feed on flowers from Mexican milkweed, *Asclepias curassavica* (AC), that were untreated (C), treated with label rate (1X), or twice label rate (2X) of soil-applied imidacloprid (Marathon 1%G) or that were force-fed 30% syrup syrup (S) containing 0 ppb (C), 15 ppb (1X), or 30 ppb (2X) imidacloprid.

**Fig 4:**
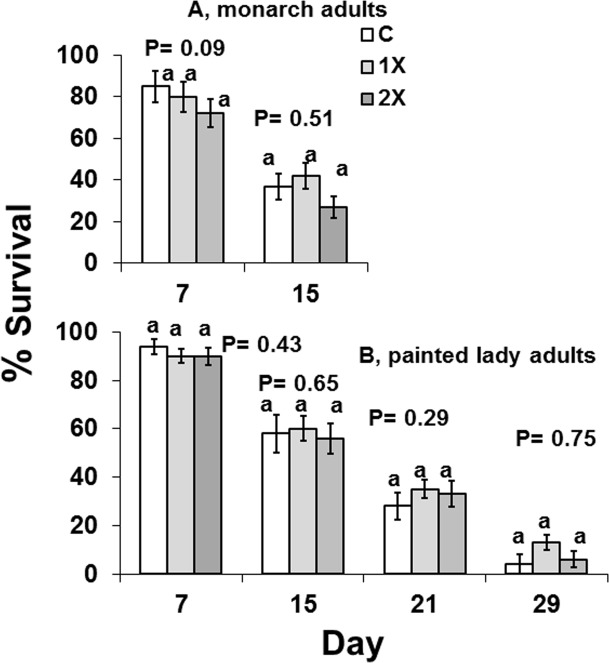
Survival of two species of adult butterflies that were free-ranging and allowed to feed on flowers from Mexican milkweed, *Asclepias curassavica*, that were untreated (C), treated with label rate (1X), or twice label rate (2X) of soil-applied imidacloprid (Marathon 1%G) for *Danaus*, monarch butterflies at days 7 and 15 and for *Vanessa*, painted lady butterflies at days 7, 15, 21, and 29.

**Fig 5:**
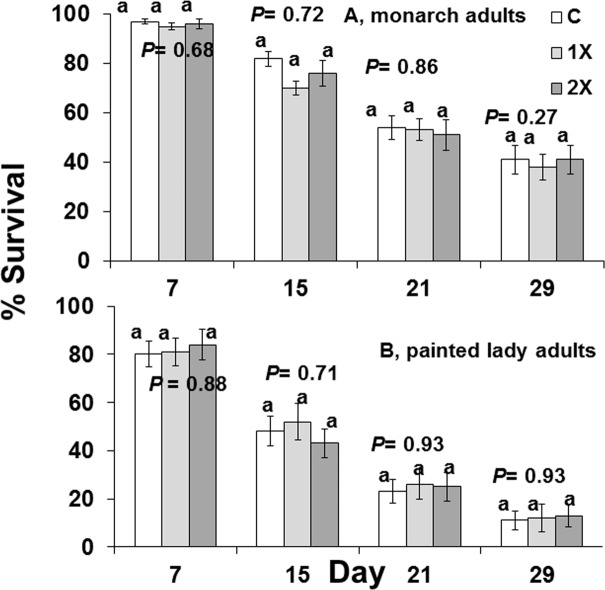
Survival at days 7, 15, 21, 29 of two species of adult butterflied that were force-fed 30% syrup containing 0 ppb (C), 15 ppb (1X), or 30 ppb (2X) imidacloprid.

Imidacloprid did not reduce the survival of free-ranging and force-fed butterflies (Fig. 3, Table 6). Monarch adults, *Danaus*, lived longer when fed 30% syrup containing imidacloprid than when free-ranging (Fig. 4, free ranging monarch survival, day 7, F = 2.77; df = 2, 29; P = 0.09; day 15, F = 0.70; df = 2, 29; P = 0.51; free ranging painted lady survival, day 7, F = 0.43; df = 2, 39; P = 0.43; day 15, F = 0.29; df = 2, 39; P = 0.65; day 21, F = 0.70; df = 2, 39; P = 0.29; day 29, F = 0.04; df = 2, 39; P = 0.75; Fig. 5, force-fed monarch survival, day 7, F = 0.39; df = 2, 57; P = 0.68; day 15, F = 0.34; df = 2, 57; P = 0.72; day 21, F = 0.15; df = 2, 57; P = 0.86; day 29, F = 0.10; df = 2, 57; P = 0.27; force fed-painted lady survival, day 7, F = 0.13; df = 2, 27; P = 0.88; day 15, F = 0.35; df = 2, 27; P = 0.71; day 21, F = 0.07; df = 2, 27; P = 0.93; day 29, F = 0.08; df = 2, 27; P = 0.93).

**Table 6:** Survival of two species of adult butterflies that were free-ranging and allowed to feed on flowers from Mexican milkweed, *Asclepias curassavica*, (AC), that were untreated (C), treated with label rate (1X), or twice label rate (2X) of soil-applied imidacloprid (Marathon 1%G) or that were force-fed 30% syrup syrup (S) containing 0 ppb (C), 15 ppb (1X) and 30 ppb (2X) imidacloprid.

	Coef	Exp (coef)	SE (coef)	*P* value
***Danaus***, **Monarchs fed treated sugar syrup**				
1X-C	0.29	1.33	0.19	0.2958
2X-C	0.19	1.21	0.19	0.5817
2X-1X	-0.09	0.91	0.19	0.8762
***Danaus*, Monarchs free-ranging on flowering**				
1X-C	0.04	1.04	0.12	0.9416
2X-C	0.19	1.21	0.12	0.2827
2X-1X	0.15	1.16	0.12	0.4594
***Vanessa*, Painted Ladies fed treated sugar syrup**				
1X-C	-0.03	0.97	0.20	0.9915
2X-C	-0.09	0.91	0.21	0.901
2X-1X	-0.06	0.94	0.21	0.9484
***Vanessa*, Painted Ladies free-ranging on flowering**				
1X-C	0.13	1.14	0.18	0.7373
2X-C	0.04	1.04	0.18	0.9762
2X-1X	-0.10	0.91	0.18	0.8548

Cox proportional hazards model with a random effect for cage, and stratified by replicate, α = 0.05.

Imidacloprid did not reduce the fecundity and egg hatch of free-ranging and force-fed butterflies (free-ranging butterflies, monarch fecundity no data; painted lady fecundity, C = 545.5 ± 191.2, 1X = 354.0 ± 87.5, and 2X = 325.6 ± 88.9; F = 0.15; df = 2, 27; P = 0.8650; painted lady % egg hatch, C = 63.7 ± 3.3, 1X = 63.5 ± 5.9, and 2X = 62.3 ± 4.0; F = 0.03; df = 2, 27; P = 0.9668; force-fed butterflies, monarch fecundity, C = 437.5 ± 51.8, 1X = 433.6 ± 57.3, and 2X = 385.3± 56.5; F = 0.79; df = 2, 57; P = 0.4618; monarch % egg hatch, C = 45.9 ± 5.5, 1X = 42.3 ± 5.9 and 2X = 41.4 ± 5.8; F = 1.23; df = 2, 56; P = 0.3029; painted lady fecundity, C = 260.4 ± 53.6, 1X = 278.0 ± 32.8, and 2X = 270.2 ± 44.6; F = 0.06; df = 2, 27; P = 0.9411; painted lady % egg hatch, C = 70.3 ± 4.0, 1X = 65.8 ± 4.4, and 2X = 76.8 ± 3.7; F = 1.75; df = 2, 27; P = 0.1960).

### Butterflies: Larval Survival

**Fig 6:**
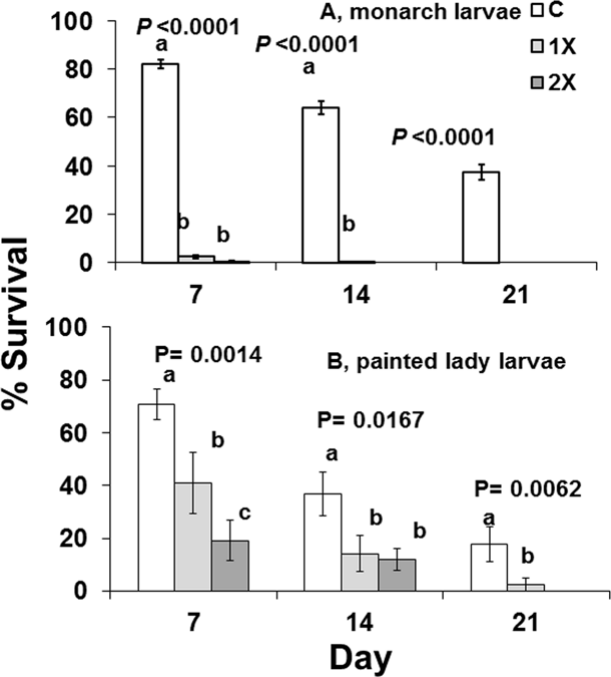
Survival at days 7, 14, and 21 of two species of larval butterflies that were free-ranging on host plants that were untreated (C), treated with label rate (1X), or twice label rate (2X) of soil-applied imidacloprid (Marathon 1%G). *Danaus*, monarch butterflies were fed Mexican milkweed, *Asclepias curassavica*, and *Vanessa* painted lady butterflies were fed globe thistle, *Echinops ritro*.

Survival of monarch, *Danaus*, and painted lady, *Vanessa*, larvae fed 1X and 2X imidacloprid-treated plants was significantly reduced by day 7 compared to controls (Fig. 6). Few monarch larvae survived after 7 days. By day 14 painted lady larval survival was 40% on controls, 20% on 1X, and 19% on 2X treatments. Percentage pupation of painted lady larvae was 22.3 ± 8.0% on controls, 2.5 ± 2.5% on 1X, and 0% on 2X treatments. (monarch larval survival, day 7, F = 631.1; df = 2, 147; P = 0.0001; day 14, F = 620.4; df = 2, 147; P = 0.0001; day 21, F = 200.5; df = 2, 147; P = 0.0001; painted lady larval survival, day 7, F = 8.27; df = 2, 33; P = 0.0014; day 14, F = 4.71; df = 2, 33; P = 0.0167; day 21, F = 6.04; df = 2, 33; P = 0.0062).

## Discussion

The two different residue determination methods produced statistically similar results (Table 1). In addition, imidacloprid residue extracted from whole flowers of two different plant species was similar (1X, 6 ppm both milkweed and buckwheat and 2X, 10.4 ppb milkweed and 12.3 ppb buckwheat), adding validity to our residue data [47] (Tables 2, 3). These data show that greenhouse/nursery rates of imidacloprid (300mg/10 L pot) result in much higher amount of residue compared to residues from seed treatments (<10 ppb in pollen and nectar from canola 0.05 or corn 1.2 mg AI/seed) [13,16] or agricultural rates [29] (122 ppb in pollen of pumpkin from a crop rate of 4 mg AI /sq ft (939/ sq cm)). Data demonstrated that soil-applied imidacloprid was translocated to pollen and nectar and reduced survival of 3 out of 4 species of lady beetles. Lady beetles did not avoid feeding on imidacloprid treated flowers as there was not a significant difference between 1X-1X, 1X-C, 2X-C, and 2X-2X treatments.

In previous studies in our laboratory, the parasitoid, *Anagyrus pseudococci* [47] and the green lacewing, *Chrysoperla carnae* [48] had altered behaviors, such as trembling and lack of coordination, and reduced survival when feeding on flowers from Mexican milkweed and buckwheat plants treated with a 1X and 2X greenhouse/nursery rate of soil-applied imidacloprid. The pink lady beetle, *Coleomegilla maculata*, when confined in mesh cages on flowers on plants treated with a 1X rate showed reduced survivorship on sunflower and reduced movement on 3 plant species [1] (sunflower, *Helianthus annus* ‘Big Smile’; chrysanthemum, *Chrysanthemum morifolium* ‘Pelee’; and dandelion, *Taraxacum officinale*).

Imidacloprid did not appear to reduce survival of adult monarchs or painted lady butterflies that were either free-ranging or force-fed. Force-fed butterflies were fed 0 ppb, 15 ppb (1X) and 30 (2X) ppb imidacloprid, which is a substantially lower concentration than what free-ranging butterflies encountered when feeding on flower residues of 6,030 ppb (1X) and 10,400 ppb (2X). Imidacloprid may not alter survival in adult butterflies, as they may not metabolize the insecticide, instead excreting it unchanged, as was shown for phytochemicals present in the host plant of *Papilio glaucus* [49] and *Orgyia leucostigma* [50]. Survival for adult monarch butterflies in force-fed imidacloprid treatments was higher than free-ranging butterflies, but painted lady butterflies survived similarly in both studies. It is possible that monarch butterflies could not forage adequately in cages, perhaps due to interference with cage walls, as monarch are twice the size of painted lady butterflies.

Larval painted ladies and monarchs had significantly lower survival when feeding on leaves treated with 1X and 2X greenhouse/nursery rates compared to controls. Recent studies demonstrate that volunteer plants growing near seed-treated crops can contain imidacloprid residues [51], which may harm butterfly larvae feeding on volunteer plants. There is growing concern that seed treatments have no economic benefit to plants and may pose an environmental risk to beneficial insects. A review of the effectiveness of seed treatments reported that of the 19 scientific peer-reviewed studies that looked at whether seed treatments increased yields, 8 found no improvement, and 11 said results were inconsistent [54].

The potential risk of mortality from imidacloprid is higher for monarch larvae feeding on milkweed plants near seed-treated crops, due to the ubiquitous use of seed-treated crops, the neonicotinoid insecticide dust drift at planting, and the accumulation of neonicotinoids in soil [13, 53]. A few years ago, the risk of pollen from transgenic corn landing on milkweed plants and killing monarch butterflies was critically evaluated by numerous researchers after data demonstrated 56% mortality in monarch larvae fed for 4 days on Mexican milkweed leaves dusted with one type of transgenic corn pollen [52]. Newer types of transgenic corn do not cause the same mortality on monarch larvae [53]. However, the risk to monarchs that feed on milkweeds growing near seed-treated crops needs to be evaluated.

Landscape rates of imidacloprid may result in high imidacloprid residues in flowers. The initial research performed by USDA APHIS to understand the effects of imidacloprid trunk injections on flowers, found that maple, *Acer* spp., and horse chestnut, *Aesculus hippocastanum*, flowers collected from trees that were trunk injected with imidacloprid 10–12 months earlier had residues of 130 ppb in one sample and 30–99 ppb in 5 samples. The report went on to discuss the potential of 130 ppb to cause bee mortality [55]. Eucalyptus trees treated with an imidacloprid soil injection (Merit 75WP label rate, Bayer CropScience, Research Triangle Park, NC) at 5 months pre-bloom expressed 660 ppb imidacloprid in nectar [56], levels that killed two beneficial wasp parasitoids, the braconid larval parasitoid, *Syngaster lepidus* (oral LC50 288 ppb imidacloprid) and the encyrtid egg parasitoid, *Avetianella longoi* (oral LC50 212 ppb imidacloprid). This is similar to the oral LC50 for honey bees of 192 ppb imidacloprid [57]. In 2013 in Oregon, linden trees (*Tilia* spp.) sprayed with dinotefuran (Safari 20 SG, Valent, Walnut Creek, CA) at flowering caused mortality of 50,000 bumble bees [58]. Turf treated with clothianidin (Arena 50 WDG, Valent, Walnut Creek, CA) resulted in residues of 171 ppb in flowers, residue levels that reduced colony health and foraging of the bumble bee *Bombus impatiens* [59]. These studies provide evidence that systemic neonicotinoid insecticides used in urban, residential landscapes can be translocated to pollen and nectar at sufficient levels to alter behavior and later cause mortality in bees and beneficial insects.

Imidacloprid has been shown to reduce foraging and colony health of bees. The actual estimated oral imidacloprid LD50 for foraging honey bees is 185 ppb [57] and 192 ppb [60]. Oral toxicity of imidacloprid to honey bees was 370 ppb at 72 h, while the olefin metabolite was more toxic (290 ppb) and the hydroxy metabolite less toxic (2,060 ppb) compared to imidacloprid [61,62]. Bayer Chemical researchers demonstrated that there was no effect on honey bees at <20 ppb [63] while at levels >20 ppb behavior was changed, as measured by a reduction in recruitment to food sources [63]. Imidacloprid reduced the orientation of honey bees at 25 ppb [64]. Foraging bees reduced their visits to feeders containing imidacloprid-treated syrup at 6 ppb [64] and 50 ppb [64]. Reduction in recruitment was postulated as a result of decrease in effectiveness of dances at the hive to recruit bees [65].

In field studies, honey bee foraging was reduced at 15 ppb imidacloprid [66], 5 ppb clothianidin [67], and 67 ppb thiamethoxam [68]. Foraging was reduced at 10 ppb imidacloprid for *Bombus terrestris* [69] and 30 ppb imidacloprid for *B*. *impatiens* [71]. Whitehorn et al. 2012 [72] showed that queenright colonies of *B*. *terrestris* fed 0.7 and 1.4 ppb imidacloprid in sugar syrup for two weeks in the lab and then monitored in the field for 6 weeks, could not recover from imidacloprid effects. Colony weight was lower by 8% and 12% and queen production was lower by 85% and 90%, respectively, compared to controls. *Bombus impatiens* workers fed 5 ng/bee (50 ppb) imidacloprid and then moved away from their nests, were impaired and lost their ability to orient to landmarks [73]. Gill et al. 2012 [70] found that *B*.*terrestris* fitted with RFID (radio frequency identification tags) and fed 10 ppb imidacloprid in sugar syrup for 4 weeks had significantly more workers (50%) that did not return to the colony. Worker foraging performance, particularly pollen collecting efficiency, was significantly reduced which led to increased colony demand for food as shown by increased worker recruitment to forage and less time spend on brood care. In the field, imidacloprid seed-treated sunflowers reduced return of *B*. *terretris* by 10% [74]. Larson et al. 2013 [59] found that queenright colonies of *B*. *impatiens* did not avoid foraging on clothianidin-treated clover (171 ppb flowers) and showed reduced foraging activity and increased worker mortality in the hives within 5 days. Colonies showed a trend for fewer workers and males, no queen production, reduced number of wax pots, and reduced colony weight compared to controls. Reduced colony weight is related to worker foraging and behavior.

The European Union’s Food Safety Authority review paper on the risk of neonicontinoid insecticides identified a deficit of studies on residue in flowers of crops and landscape plants [16]. The Mexican milkweed residue data presented in this paper and the buckwheat residue data presented in our 2007 paper showed similar amounts of imidacloprid in flowers from a 1X and 2X greenhouse/nursery application rates [47]. Thus, greenhouse/nursery application rates can result in 793 to 1,368 times more imidacloprid in a plant compared to a seed treatment (7.6 ppb imidacloprid in canola nectar). Research in our lab showed that these greenhouse/nursery application rates killed adult lacewing [48], adult parasitic wasps [47], and adult lady beetles 1confined to plants [1]. In this study 3 of 4 species of ladybeetles had reduced survival from a greenhouse/nursery application rate of imidacloprid. In addition, larval survival of two butterfly species, *Danaus plexippus* and *Vanessa cardui*, was reduced on imidacloprid treated plants. Consequently, the use of systemic, neonicotinoid insecticides, such as imidacloprid, increased the risk of mortality in beneficial insects and their use may not be compatible with conserving pollinators and biological control organisms, which are vital components of IPM programs. Reducing neonicotinoid use on flowering plants grown in greenhouse, nursery, and landscapes merits additional research and advocacy.
